# New Insight on Carbon Dioxide‐Mediated Hydrogen Production**

**DOI:** 10.1002/open.202100262

**Published:** 2022-04-03

**Authors:** Antoni W. Morawski, Ewelina Kusiak‐Nejman, Iwona Pełech, Katarzyna Ćmielewska, Daniel Sibera, Piotr Staciwa, Agnieszka Wanag, Marcin Gano, Ewa Ekiert, Joanna Kapica‐Kozar, Kordian Witkowski, Urszula Narkiewicz

**Affiliations:** ^1^ Department of Chemical Inorganic Technology and Environment Engineering Faculty of Chemical Technology and Engineering West Pomeranian University of Technology in Szczecin Pułaskiego 10 70-322 Szczecin Poland; ^2^ Department of General Civil Engineering Faculty of Civil and Environmental Engineering West Pomeranian University of Technology in Szczecin Piastów Ave., 50a 70-311 Szczecin Poland; ^3^ Department of Chemical Organic Technology and Polymeric Materials Faculty of Chemical Technology and Engineering West Pomeranian University of Technology in Szczecin Piastów Ave., 42 71-065 Szczecin Poland

**Keywords:** carbon dioxide fixation, carbon storage, hydrogen, photochemistry, photolysis

## Abstract

A new approach to hydrogen production from water is described. This simple method is based on carbon dioxide‐mediated water decomposition under UV radiation. The water contained dissolved sodium hydroxide, and the solution was saturated with gaseous carbon dioxide. During saturation, the pH decreased from about 11.5 to 7–8. The formed bicarbonate and carbonate ions acted as scavengers for hydroxyl radicals, preventing the recombination of hydroxyl and hydrogen radicals and prioritizing hydrogen gas formation. In the presented method, not yet reported in the literature, hydrogen production is combined with carbon dioxide. For the best system with alkaline water (0.2 m NaOH) saturated with CO_2_ under UV‐C, the hydrogen production amounted to 0.6 μmol h^−1^ during 24 h of radiation.

## Graphical abstract

**The capture of hydroxyl radicals** by adsorbed CO_2_ in alkaline water according to the reactions ^⋅^OH+CO_3_^2−^→OH^−^+CO_3_^⋅−^ and ^⋅^OH+HCO_3_^−^→OH^−^+HCO_3_^⋅−^ is shown to be crucial for the production of hydrogen during the photolysis of water under UV light. The production of up to 0.6 μmol h^−1^ of hydrogen in 24 h with CO_2_‐saturated alkaline water (0.2 m NaOH) under UV‐C radiation represents the best results.

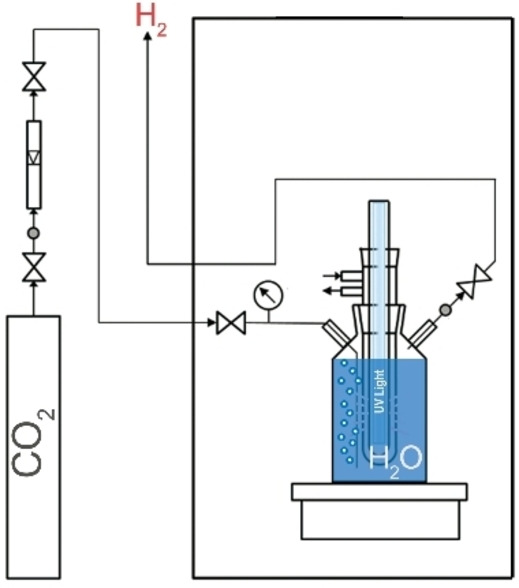

## Introduction

The rapid development of the world economy and the growth in the number of people are leading to an increased demand for energy. According to the International Energy Agency, the energy demand is estimated to increase by 30 % by 2040.[^1^, ^2^] Simultaneously, the scenario proposes reducing CO_2_ emissions by more than 40 % in 2015–2040. Currently, the EU Commission reports that global fossil CO_2_ emissions still increased by 0.9 % in 2019 to a total of 38 Gt CO_2_. Also, CO_2_ emissions per capita have increased by about 15 % from 4.26 t CO_2_/capita/year to 4.93 t CO_2_/capita/year between 1990 and 2019.^[3]^ Therefore, to reduce CO_2_ emissions, the search for new technologies with zero CO_2_ emissions is still attractive. One of the directions is the splitting of water to obtain hydrogen fuel.

The milestone was the experiment conducted by Fujishima and Honda, in which water was more effectively split into hydrogen and oxygen when a p‐type semiconductor electrode containing TiO_2_ was irradiated with visible light.^[4]^

Since then, the photocatalytic research direction related to the decomposition of water (water splitting) into oxygen and hydrogen has developed intensively. Almost 3,000 publications can be found in the Scopus literature database under the heading *“water photocatalytic decomposition*”. One of the most important recent works describes a very active photocatalyst based on modified TiO_2_ with 1.5 % by mass of copper, which shows the hydrogen evolution rate of 101.7 mmol ⋅ g^−1^ ⋅ h^−1^ under simulated solar light irradiation, which is the highest among those described so far in the literature.^[5]^ The stability of the activity for 380 days with an apparent quantum efficiency of 56 % at 365 nm was also proven.

Then, the decomposition of water was combined with the reduction of CO_2_ to obtain hydrogen, carbon monoxide, and useful hydrocarbons. In this case, too, almost 9,000 works were published. The main products of the photocatalytic reduction of carbon dioxide can be hydrogen, methane, carbon monoxide, methanol, and other hydrocarbons. The selectivity of the reaction depends on many factors, such as the type of photocatalyst, its crystallinity, introduced modifying elements, type of carrier, and others.^[6]^

In the publication of Soltani et al.,^[7]^ selective photocatalysts for hydrogen production with reduction of CO_2_ were developed. Obtaining high selectivity to hydrogen is controversial because the hydrogen obtained from the decomposition of water is used immediately to reduce carbon dioxide, and it is challenging to implement in the photocatalytic reduction of CO_2_. Additionally, there are the costs of the photocatalyst and the operational costs associated with separating the photocatalyst from the product mixture.

This paper proposes a simple and selective method of obtaining hydrogen without using a photocatalyst. An alkaline sodium hydroxide solution was used to perform the experiments because its role and action in the high‐temperature production processes of hydrogen have been presented and described.^[8]^ The obtained amounts of hydrogen are at the micromolar level, which is often found in non‐selective photocatalytic methods.

This work takes advantage of the fact that the energy of radiation with a wavelength in the UV range is sufficient to excite the H−OH bond in water, which is 498 kJ mol^−1^. For example, a quantum of radiation with a wavelength of 200 nm has an energy of about 590 kJ mol^−1^.^[9]^ Another fact that was of great importance for these studies was using the inactivation capacity of ⋅OH hydroxyl radicals by HCO_3_^−^ and CO_3_^2−^ ions, formed when water is saturated with carbon dioxide,^[10]^ act as radical scavengers.

## Results and Discussion

We have conducted some preliminary and comparative experiments. In the first experiment, 500 cm^3^ of water not saturated with gaseous CO_2_ was added to the reactor. Then, the water was irradiated with a UV‐C TQ 150 lamp. After 6 h of irradiation, no hydrogen was found in the reactor. Next, the analogical test was carried out for 0.2 m solution of NaOH, which was not saturated with gaseous CO_2_. Same as before, no hydrogen was obtained due to irradiating the reactor‘s content with UV radiation.

**Figure 1:**
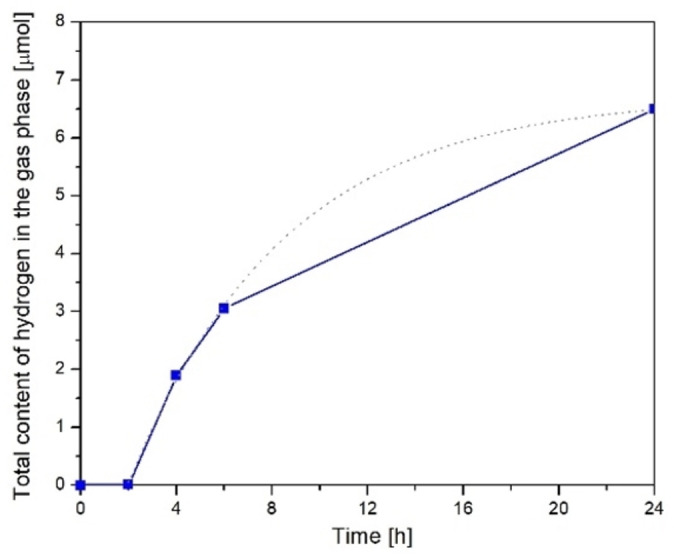
Hydrogen production from water saturated with CO_2_ under UV‐C TQ lamp radiation.

In the second experiment, 500 cm^3^ of water were saturated with CO_2_ gas for 16 h. Then the UV‐C lamp was turned on. The hydrogen content was analysed in the gas phase above the water surface. The results obtained are shown below in Figure 1.

The measured hydrogen content gradually increased and reached 6.4 μmol after 24 h of irradiation. These changes are not linear throughout the experimental period. After 2–6 h of irradiation, the hydrogen formation rate increased to 0.75 μmol h^−1^ and then decreased to about 0.2 μmol h^−1^. The average reaction rate of hydrogen production over 0–24 h can be estimated at 0.266 μmol h^−1^. At the same time, an increase in the oxygen concentration in the reactor was also observed.

**Figure 2:**
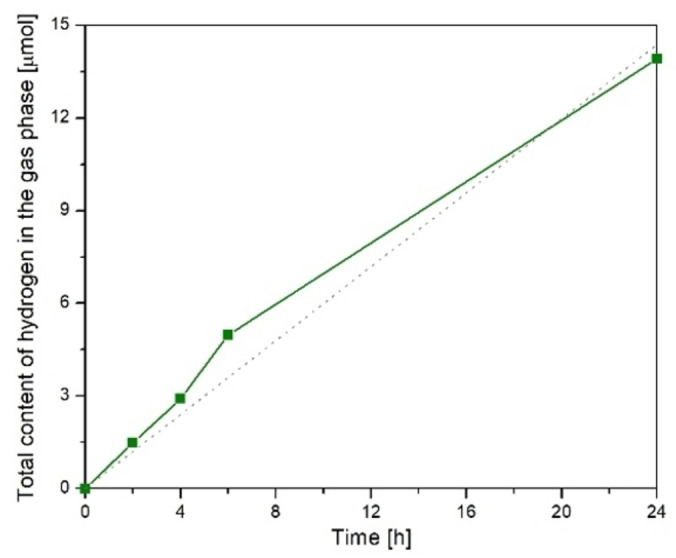
Hydrogen production by alkaline water (0.2 m NaOH) saturated with CO_2_ under UV‐C TQ lamp radiation.

**Figure 3:**
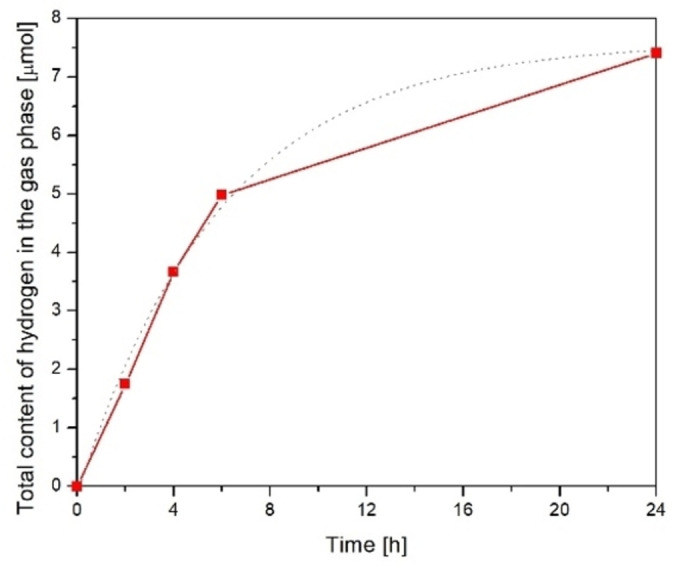
Hydrogen production from alkaline water (0.2 m NaOH) saturated with CO_2_ under UV‐A TQ 150 Z3 mercury lamp radiation with lower UV‐C component.

The next step of the experiment was as follows: 500 cm^3^ of alkaline water containing 0.2 m NaOH was saturated with CO_2_ for 16 h. The hydrogen content in the gas phase above the solution was measured during UV‐C irradiation with the TQ lamp. The results obtained are shown in Figure 2. A comparative experiment was performed using a UV‐A TQ 150 Z3 mercury lamp with a lower UV‐C content (Figure 3).

The results presented in Figure 2 show an almost straight line dependence of the hydrogen concentration on time, and the reaction rate can be estimated with a constant of about 0.6 μmol/h. In the first 6 h of the process, the rate was about 0.83 μmol/h.

The measured hydrogen content gradually increased and reached 7.4 μmol after 24 h of irradiation. These changes are not linear over the entire experimental period. At 0–6 h of irradiation, the rate of hydrogen formation increased to 0.83 μmol/h and then decreased to about 0.14 μmol h^−1^. The overall average reaction rate of hydrogen production over 0–24 h can be estimated to be 0.31 μmol h^−1^.

**Figure 4:**
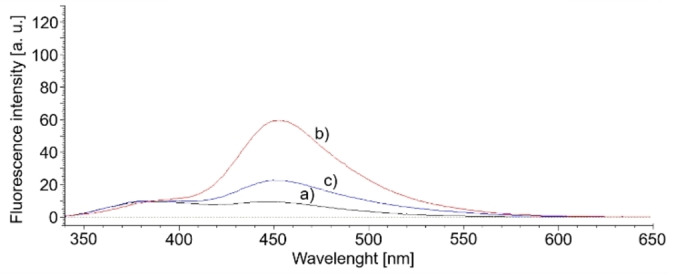
Fluorescent spectra of 7‐hydroxycoumarin obtained from: a) 10^−3^ m coumarin aqueous solution; b) 10^−3^ m coumarin aqueous solution irradiated with UV‐C lamp for 6 h; c) 10^−3^ m coumarin aqueous solution saturated with CO_2_, and then irradiated with UV‐C lamp for 6 h.

**Figure 5:**
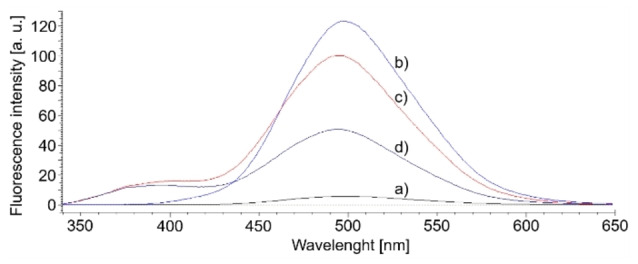
Fluorescent coumarin derivative spectra obtained from: a) 10^−3^ m coumarin solution in 0.2 m NaOH; b) 10^−3^ m coumarin solution in 0.2 m NaOH irradiated with UV‐C lamp for 6 h; c) 10^−3^ m coumarin solution in 0.2 m NaOH saturated with CO_2_, and irradiated with UV‐C lamp for 6 h; d) 10^−3^ m coumarin solution in 0.2 m NaOH saturated with CO_2_, and irradiated with UV‐A lamp for 6 h.

Table 1 summarises the coumarin‐based fluorescent products′ peak area with hydrogen production after 6 h of irradiation. It can be seen that hydrogen appeared only in samples saturated with carbon dioxide and a higher amount for samples that initially had a highly alkaline value of pH by introducing NaOH. Another interesting piece of information from Table 1 was the amount of hydroxyl radicals in the tested samples. Water saturation with carbon dioxide (sample H_2_O+coumarin+CO_2_ (UV‐C)) generated more than 2.5 times fewer hydroxyl radicals than the sample without CO_2_, which confirms the ability of carbonate and hydroxycarbonate ions to scavenge hydroxyl radicals. Almost the same behavior occurs in samples containing alkaline NaOH. The above‐discussed tendencies of hydroxyl radicals generation can be seen from Figures 4 and 5.

**Table 1:** Comparison of the intensity of the coumarin‐based fluorescent products with the production of hydrogen before and after 6 h of irradiation.

Sample name	Fluorescence peak area after 6 h of irradiation [a. u.]	Relative peak area	H_2_ production after 6 h of irradiation [μmol H_2_]
H_2_O+coumarin	1159	1.00	–
H_2_O+coumarin (UV‐C)	5313	4.58	–
H_2_O+coumarin+CO_2_ (UV‐C)	2203	1.90	3.06
H_2_O+coumarin+NaOH	1401	1.21	–
H_2_O+coumarin+NaOH (UV‐C)	10949	9.45	–
H_2_O+coumarin+NaOH+CO_2_ (UV‐C)	10085	8.70	4.97
H_2_O+coumarin+NaOH+CO_2_ (UV‐A)	5591	4.82	4.97

Water is well known to be sensitive to solar radiation.^[12]^ The products of this process are hydrogen and hydroxyl radicals:

(1) $$H{}_{2}O+h\upsilon \to H{}^{•}+{}^{•}\mathrm{OH}$$

Due to the short lifetime of the radicals, recombination of the products occurs to produce water:

(2) $$H{}^{•}+{}^{•}\mathrm{OH}\to H{}_{2}O$$

Other possible recombination pathways with a constant rate of k=4.7 m ^−1^ ⋅ s^−1^ at 25 °C^[13]^ are as follows:

(3) $${}^{•}\mathrm{OH}+{}^{•}\mathrm{OH}\to H{}_{2}O{}_{2}$$

or

(4) $$H{}^{•}+H{}^{•}\to H{}_{2}$$

Reaction (2) supports why pure water without CO_2_ saturation did not produce hydrogen gas in the first experiment during UV‐C irradiation.

If we are interested in hydrogen production, the hydroxyl radicals OH⋅ should be blocked to prevent water recombinations according to reaction (2). For this purpose, we used dissolved bicarbonate and carbonate ions formed according to the mechanism shown below. Dissolution of carbon dioxide in water leads to the formation of carbonic acid, which is in equilibrium according to the following reactions:[^10^, ^14^]

(5) $$\mathrm{CO}{}_{2}+H{}_{2}O↔H{}_{2}\mathrm{CO}{}_{3}$$

(6) $$H{}_{2}\mathrm{CO}{}_{3}↔\mathrm{HCO}{}_{3}{}^{-}+H{}^{+}$$

(7) $$\mathrm{HCO}{}_{3}{}^{-}↔\mathrm{CO}{}_{3}{}^{2-}+H{}^{+}$$

Reaction (5) dominates at pH less than 8.3, reaction (6) takes place at pH between 6–10, while reaction (7) takes place at pH from 8.3 to 11.

In our reactor, the combined saturation reactions occur as follows:

(8) $$2\;\mathrm{NaOH}+H{}_{2}O+\mathrm{CO}{}_{2}\to \mathrm{Na}{}_{2}\mathrm{CO}{}_{3}+2H{}_{2}O$$

(9) $$\mathrm{Na}{}_{2}\mathrm{CO}{}_{3}+H{}_{2}O+\mathrm{CO}{}_{2}\to 2\;\mathrm{NaHCO}{}_{3}$$

Both the bicarbonate and carbonate ions from reactions (6) and (7), respectively, are known to be fast acceptors (scavengers) of hydroxyl radicals in water^[15]^ at a constant rate: k=4.2 ⋅ 10^8^ mol^−1^ ⋅ dm^3^ ⋅ s^−1^ for CO_3_^2−^ ions, and k=1.5 ⋅ 10^7^ mol^−1^ ⋅ dm^3^ ⋅ s^−1^ for HCO_3_^−^ ions, corresponding to the following reactions:

(10) $${}^{•}\mathrm{OH}+\mathrm{CO}{}_{3}{}^{2-}\to \mathrm{OH}{}^{-}+\mathrm{CO}{}_{3}{}^{•}{}^{-}$$

(11) $${}^{•}\mathrm{OH}+\mathrm{HCO}{}_{3}{}^{-}\to \mathrm{OH}{}^{-}+\mathrm{HCO}{}_{3}{}^{•}{}^{-}$$

In reactions (10) and (11), forming hydroxyl radicals is impossible. Hydroxyl ions could react with Na^+^ to form NaOH and again with carbon dioxide, presented in reactions (8) and (9). According to the above mechanism, the split hydrogen radicals H⋅ from reaction (1) can freely participate (4) to form gaseous hydrogen H_2_.

A common feature of all reactions with the production of highly active hydroxyl radicals, especially the OH radical, is the typical value of the reaction rate constants, which is in the range 10^8^ to 10^10^ m ^−1^ ⋅ s^−1^.

In a specific reaction system, the reaction rate depends mainly on the concentration of OH radicals, which may vary depending on the various conditions of the aqueous surroundings matrices. For example, replacing oxygen with nitrogen during recombination of hydroxyl radicals lowers the rate constant by more than 2.5 times.^[16]^ Also, temperature radically influences the constant rate of recombination of hydroxyl radicals.^[17]^

The addition of small amounts of phosphorus oxides and acid to water increases the relaxation rate of OH radicals to equilibrium concentration following H_2_O photolysis. It seems to be the predominant path for OH recovery from recombination of H and OH radicals to form water. In our work, we used carbonates and hydrocarbons to achieve this goal.[^18^, ^19^]

The use of sodium hydroxide increases the concentration of bicarbonate and carbonate ions during CO_2_ saturation. In the case of our system, that is, an aqueous NaOH solution with an initial pH of around 11.5 and later saturated with CO_2_ to form Na(HCO_3_)_2_ and Na_2_CO_3_, we have a significant advantage in the concentration of sodium carbonates and bicarbonates and a high density of the solution, which determine the diffusion of low concentration radicals and their reactivity. The dominated concentration of carbonates and bicarbonates will determine the rate of the competitive reaction for capturing hydrophilic radicals, that is, bicarbonate and carbonate radicals form preferentially.

The proposed mechanism of hydrogen formation with the trapping of hydroxyl radicals acts as a pump that continuously removes hydrogen from water.

## Conclusion

An innovative approach to producing hydrogen from water under CO_2_ and UV radiation has been presented. In order to obtain hydrogen gas from water, a way to prevent the recombination of hydrogen and hydroxyl radicals formed in water during exposure to UV radiation is essential and dependent on the UV‐C radiation component. For this purpose, it is best to use bicarbonate and carbonate ions, which are formed in an alkaline environment when the water is saturated with carbon dioxide. When the pH is lowered, the hydroxyl radicals react with bicarbonate and carbonate ions, irreversibly inactivating the hydroxyl radicals.

In this way, the hydrogen radicals of H⋅ combine to form hydrogen gas. The presented method is a new and straightforward method of producing hydrogen using CO_2_ as an “extraction pump” for hydrogen from water.

## Experimental Section

**Figure 6:**
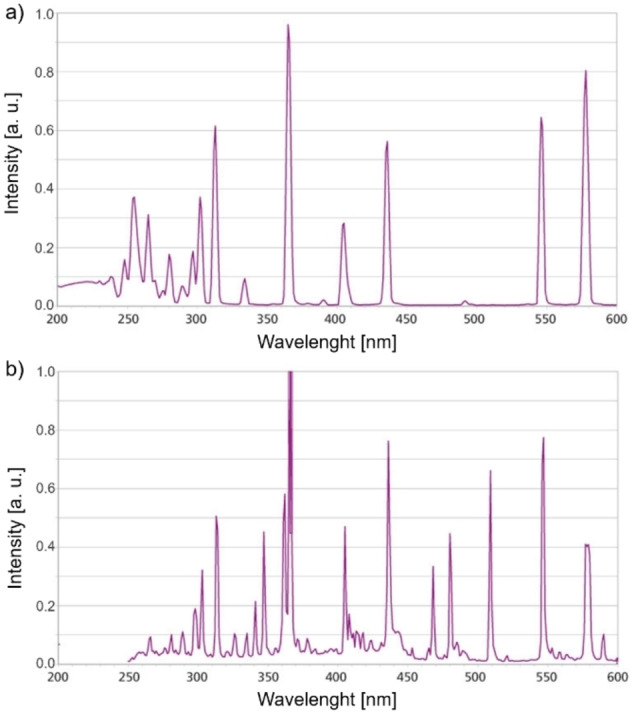
Emission spectra of lamps used in this study: a) UV‐C TQ 150; b) UV‐A TQ 150 Z3.

The experiments were performed in a cylindrical quartz reactor with a working volume of 766 cm^3^. A medium‐pressure mercury lamp TQ 150 (Heraeus, Germany) with a power of 150 W and UV‐C radiation (or a mercury lamp UV‐A TQ 150 Z3 with lower UV‐C content) was located in a cooler inside the reaction vessel. The spectra of the lamps are shown in Figures 6a and 6b.

**Figure 7:**
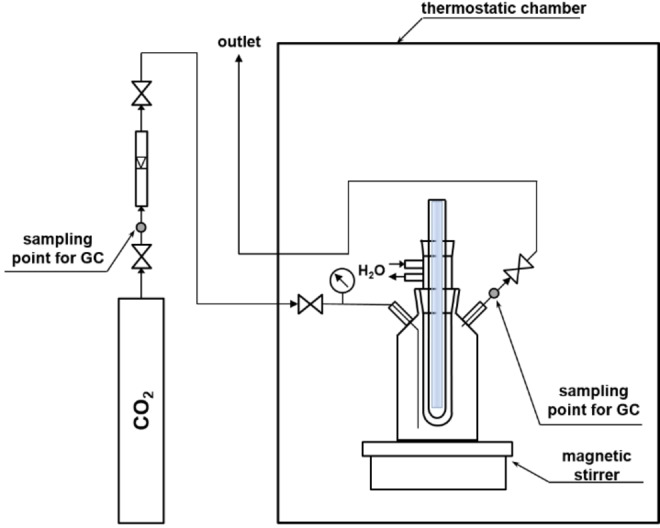
Schematic of the reactor for liquid phase processes.

The cooler was supplied with water during the experiments. The reactor was sealed in a thermostatic chamber to maintain stable temperature conditions and eliminate light sources. All experiments were performed at 20 °C. The initial pressure in the reactor was 0.2 bar. The scheme of the reactor is shown in Figure 7.

500 cm^3^ of distilled water or 500 cm^3^ of 0.2 m sodium hydroxide solution (prepared from reagent grade NaOH; POCH, Poland) was poured into the reactor, and then the system was saturated with CO_2_ by bubbling for 16 h. The reactor was then tightly sealed, and the lamp and the magnetic stirrer were turned on. The content of the reactor was irradiated for 24 h. The gas phase above the liquid surface was analyzed by gas chromatography after 0, 2, 4, 6, and 24 h.

All gas‐phase composition analyses were performed using a chromatographic method using SRI 310C gas chromatograph (SRI Instruments, USA), equipped with packed Porapak Q 100/120 column and HID detector (Helium Ionization Detector). The analyses were performed under isothermal conditions at 60 °C. Helium was used as the carrier gas. The gas flow through the column was 60 cm^3^ min^−1^, while the volume of the gas sample studied was 1 cm^3^. The hydrogen content in the gas phase volume was calculated from the calibration curve in the subsequent measurements.

Determination of hydroxyl radicals was carried out using coumarin as a fast radical scavenger to obtain 7‐hydroxycoumarin^[20]^ or other coumarin‐based fluorescent products (excited at 332 nm, with maximum emission at ca. 500 nm). 10^**−**3^ m coumarin aqueous solution was irradiated in the reactor for 6 h. The amount of 7‐hydroxycoumarin and additional fluorescent coumarin derivative obtained in the tested solutions were measured by recording fluorescence spectra using a fluorescence spectrometer F‐2500FL (Hitachi, Japan) with 332 nm excitation (PMT voltage 400 V, scan speed 300 nm ⋅ min^**−**1^, EX/EM slits:2.5/5.0 nm).

## Conflict of interest

The authors declare no conflict of interest.

1

## Supporting information

As a service to our authors and readers, this journal provides supporting information supplied by the authors. Such materials are peer reviewed and may be re‐organized for online delivery, but are not copy‐edited or typeset. Technical support issues arising from supporting information (other than missing files) should be addressed to the authors.

Supporting Information

## Data Availability

The data that support the findings of this study are available from the corresponding author upon reasonable request.
