# Supplementary material for: New Insight on Carbon Dioxide‐Mediated Hydrogen Production
Source: ChemistryOpen. 2022 Apr 3;11(4):e202100262. doi: 10.1002/open.202100262 (PMC8977505; doi:10.1002/open.202100262)
Supplement: Supplementary file 1 — Supporting Information [file OPEN-11-e202100262-s001.pdf]

Author Contributions

A.M. Conceptualization:Lead; Writing – original draft:Lead
E.K.-N. Methodology:Equal
I.P. Methodology:Equal
K.C. Data curation:Equal; Investigation:Lead; Writing – original draft:Equal
D.S. Investigation:Equal
P.S. Investigation:Equal
A.W. Investigation:Equal
M.G. Investigation:Equal
E.E. Investigation:Equal
J.K.-K. Investigation:Equal
K.W. Investigation:Equal
U.N. Project administration:Lead; Writing – review & editing:Equal
